# Laparoscopic Management of a Strangulated Hiatal Hernia: A Case Report

**DOI:** 10.7759/cureus.84731

**Published:** 2025-05-24

**Authors:** Abed AlRaouf Kawtharani, Zahraa Zeiter, Ghiwa Soufan, Houssein Haidar Ahmad, Bilal Hotayt

**Affiliations:** 1 Gastroenterology and Hepatology, Lebanese University Faculty of Medicine, Hadat, LBN; 2 Internal Medicine, Lebanese University Faculty of Medicine, Hadat, LBN; 3 General Surgery, Al Rassoul Al-Aazam Hospital, Beirut, LBN; 4 Gastroenterology and Hepatology, Al Rassoul Al-Aazam Hospital, Beirut, LBN

**Keywords:** hiatal hernias, laparoscopic hernia repair, laparoscopic technique, paraesophageal hiatal hernia, stomach strangulation

## Abstract

Strangulation of type III hiatal hernia is a rare complication that needs immediate surgical intervention to prevent lethal outcomes. The report presents a case of an 85-year-old male who presented to the emergency department with symptoms of severe epigastric pain and multiple episodes of vomiting. Diagnosis was made clinically in addition to radiological imaging, including chest radiography and CT scan that revealed a large mixed type of hiatal hernia, with the majority of the stomach sliding into the thoracic cavity. Management was via urgent surgical repair. Hiatal hernia is a pathological condition characterized by the herniation of abdominal contents through the esophageal hiatus of the diaphragm into the mediastinum. Symptoms vary, and complications may occur; early diagnosis with urgent surgical repair is needed for incarcerated types of hernias. This case report sheds light on the rare complication of type III hiatal hernia. It emphasizes the necessity of early surgical intervention, with the importance of keeping it in mind as a differential diagnosis in emergency department settings.

## Introduction

A hiatal hernia occurs when parts of the abdominal cavity, usually the stomach, herniate into the mediastinum through the esophageal hiatus [1]. Over 95% of hiatal hernias are classified as sliding hernias. This type occurs when the gastroesophageal junction (GEJ) shifts toward the hiatus and is categorized as type I. The other types are as follows: type II, a paraoesophageal hiatal hernia, occurs when the stomach moves alongside the esophagus into the mediastinum. Type III includes a sliding hernia and a paraoesophageal hernia, where the GEJ and part of the stomach have entered the mediastinum. Type IV occurs when the stomach and other organs, such as the spleen, colon, or small intestine, herniate into the chest [2]. Hiatal hernia is said to be 14.5-22% prevalent in Western populations [3].

According to Alsahafi et al., 28.9% of the Saudi population in a large endoscopy-based study had hiatal hernia [3]. Diagnosing a hiatal hernia can be challenging due to anatomical shifts in the esophago-gastric junction during swallowing, breathing, and movement. A thorough history and physical examination are essential, as they may uncover previously unnoticed symptoms [4]. Larger hiatal hernias are more frequently linked to obstructive symptoms such as dysphagia, vomiting, or discomfort/pain resulting from the compression of nearby organs/tissues. In extreme instances, large hiatal hernias might become incarcerated, leading to ischemia in herniated tissues and requiring urgent surgical intervention [5]. These complications, including strangulation, commonly occur in paraesophageal hernias, while they are extremely rare in sliding types of hernias. As such, incarcerated hiatal hernia is a serious condition that requires immediate surgical intervention, either via laparoscopy or laparotomy [6]. Therefore, our aim in this case report is to present a rare complication of type III hiatal hernia manifested as strangulation and to shed light on the whole process from the time of diagnosis till the final management.

## Case presentation

An 85-year-old male presented to the emergency department with a few hours’ history of severe epigastric pain and multiple episodes of coffee-ground vomiting. His past medical history was significant for hypertension and coronary artery disease, with a coronary artery bypass graft performed 15 years earlier, and a history of smoking. His current medications included a beta‐blocker, a statin, and an antiplatelet agent.

**Figure 1:**
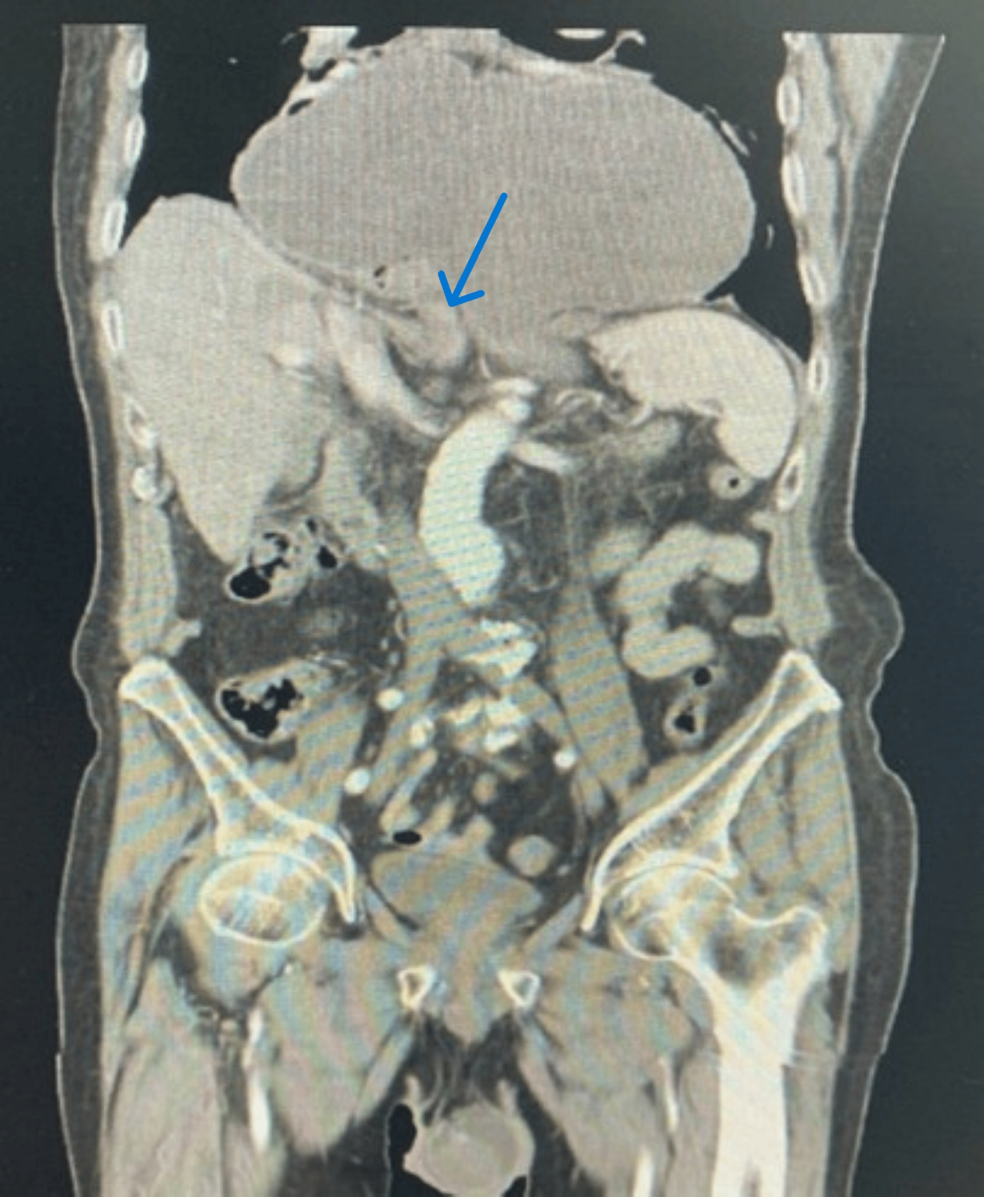
CT scan showing mixed-type incarcerated hiatal hernia with the blue arrow pointing at the pylorus CT: computed tomography

Upon arrival, the patient was alert; his blood pressure was elevated at 170/120 mm Hg. Physical examination revealed a soft abdomen with epigastric tenderness. Initial investigations, including an electrocardiogram and blood tests (troponin, lipase, and liver enzymes), were unremarkable, except for leukocytosis (Table 1). A chest X‐ray demonstrated the presence of a hiatal hernia. A CT scan of the abdomen with intravenous contrast was subsequently performed. It revealed a large mixed-type hiatal hernia, with the majority of the stomach sliding into the thoracic cavity while a portion remained in the abdominal cavity. Notably, the stomach was rotated, and the gastroduodenal junction had herniated through the esophageal hiatus, causing an obstruction that led to a distended, fluid-filled intrathoracic stomach and esophagus (Figure 1).

**Table 1:** Laboratory investigation of our patient upon presentation WBC: white blood cells, ALT: alanine transaminase, GGT: gamma-glutamyl transferase, CO2: carbon dioxide

Parameter	Patient value	Reference range
WBC	15.7 k/microL	4-11 k/microL
Hemoglobin	14.6 g/dl	13.5-18 g/dl
Platelets	208 k/microL	140-440 k/microL
ALT	17 IU/L	6-40 IU/L
GGT	37 U/l	11-50 U/l
Lipase	40 U/l	8-57 U/l
Troponin I Hs	11.3 ng/L	0-34 ng/L
CO2	24 mmol/l	20-28 mmol/l

An urgent gastroscopy was then performed to decompress the stomach and attempt an endoscopic reduction of the paraoesophageal herniation of the pylorus. Although the procedure successfully evacuated a large volume of coffee-ground fluid and minimal fresh blood was noted, multiple ischemic ulcerations were observed, and the endoscopic reduction was unsuccessful.

**Figure 2:**
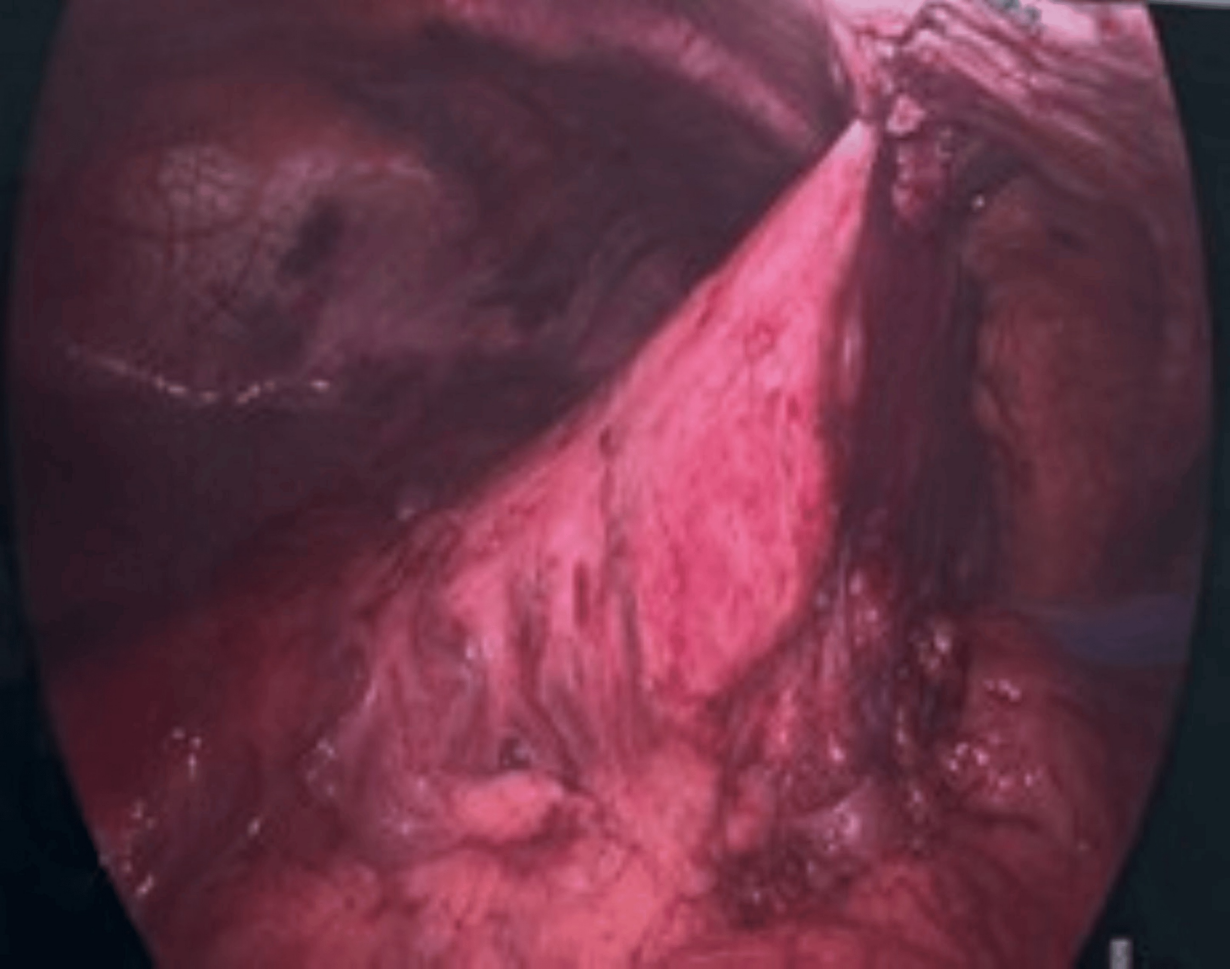
Gastropexy of the stomach to the anterior abdominal wall

Given the life-threatening nature of the condition, the patient was taken urgently to the operating room for surgical repair. Laparoscopy revealed a strangulated hiatal hernia with the entire stomach displaced into the thoracic cavity. The stomach was carefully reduced into the abdominal cavity, where ischemic changes in the fundus were noted as bruising without necrosis. A posterior cruroplasty was performed without fundoplication to avoid further compromise of the gastric blood supply, and a gastropexy was secured to the anterior abdominal wall to reduce the risk of recurrence (Figure 2). There were no intraoperative or postoperative complications.

**Figure 3:**
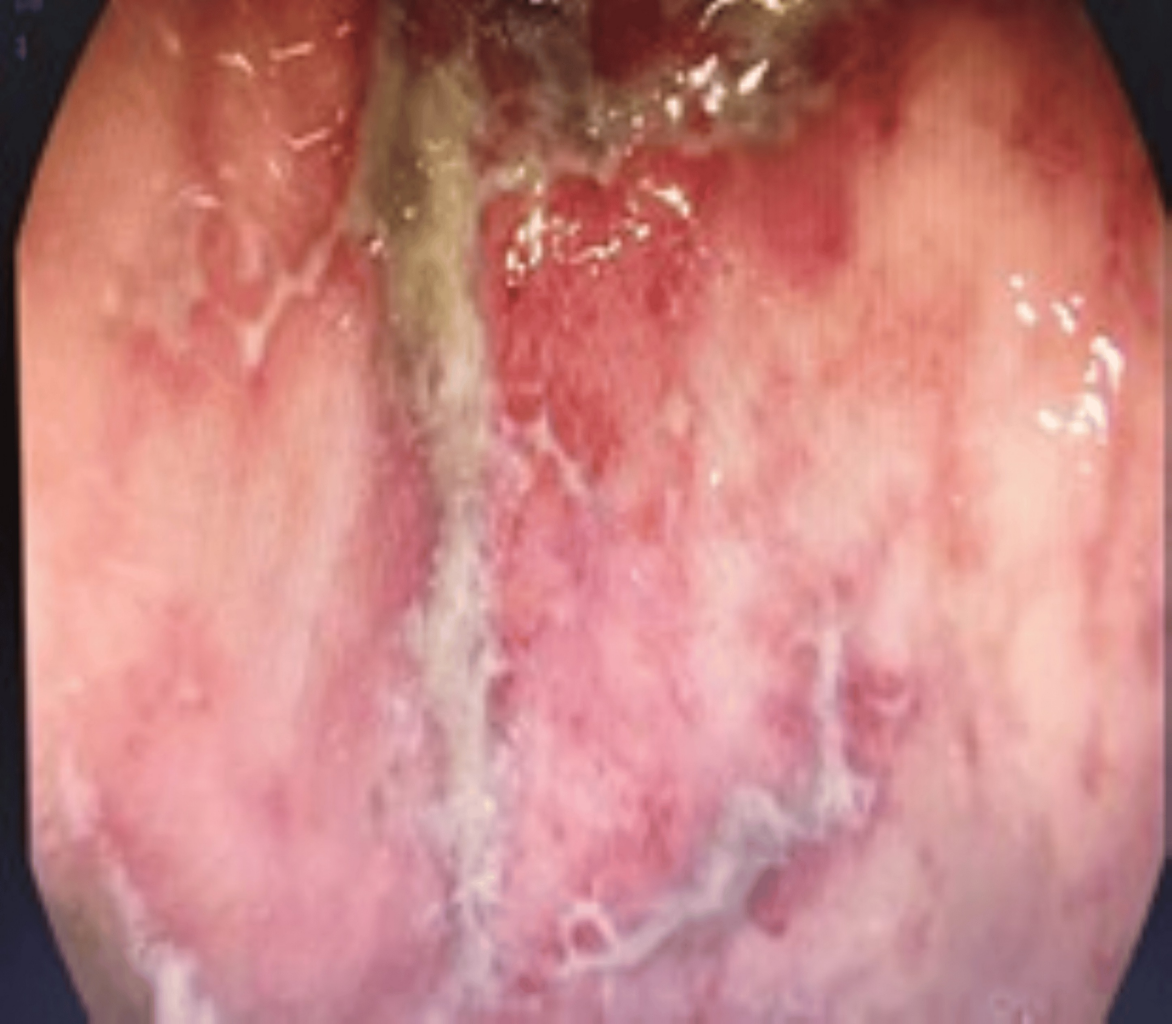
Gastroscopy showing ulcers with regenerative tissues done post-operation

**Figure 4:**
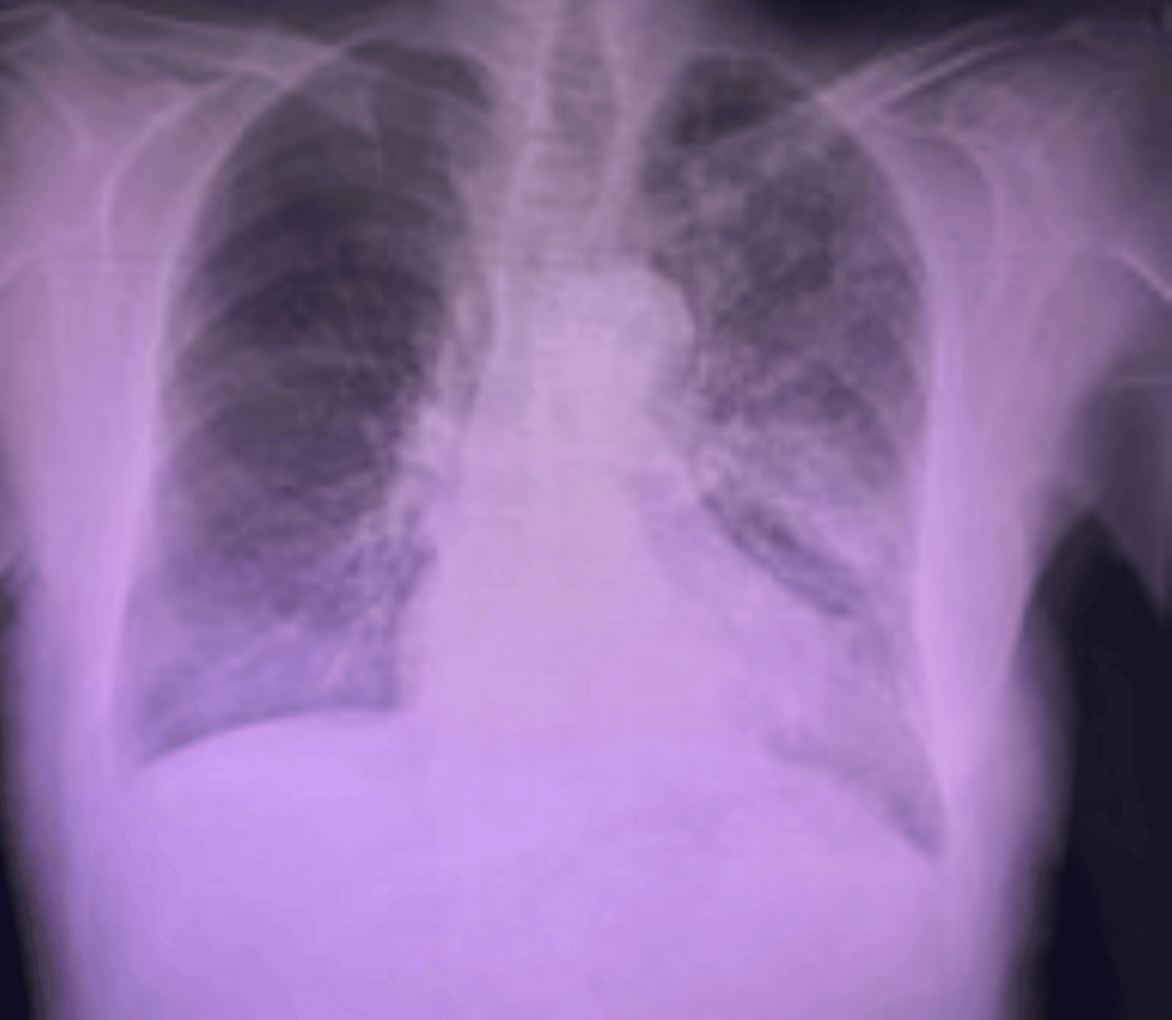
Chest X-ray done post-operation before discharge

The patient was transferred to the intensive care unit and remained intubated. Forty-eight hours postoperatively, a follow-up gastroscopy demonstrated geographic ischemic ulcers with regenerative tissue and confirmed the absence of necrosis (Figure 3). The patient was extubated on postoperative day 7, commenced on a soft diet, and tolerated it well. A follow-up chest X-ray showed the correct stomach position in the abdomen (Figure 4). He was discharged home in a stable condition 10 days after surgery.

## Discussion

Hiatal hernia, which is divided into four subtypes based on the location of the GEJ and whether additional organs are implicated, refers to the upward displacement of the stomach or other organs into the thoracic cavity via the diaphragmatic hiatus [7]. Although there are only a limited number of studies exploring the link between molecular and cellular modifications of the diaphragm and the development of hiatal hernias, three main pathogenic theories seem to emerge: (I) elevated intra-abdominal pressure pushes the GEJ upward into the thoracic cavity; (II) shortening of the esophagus caused by fibrosis or excessive stimulation of the vagus nerve moves the GEJ into the thorax; and (III) the GEJ shifts into the chest as a result of an enlarging diaphragmatic hiatus due to congenital or acquired molecular and cellular alterations, such as the irregularities in collagen type 3 alpha 1 [8].

Age and obesity are known risk factors for hiatal hernia, along with conditions like numerous pregnancies, esophageal surgery, and partial or complete gastrectomy, which our patient had not undergone [9]. Common symptoms of hiatal hernia include gastric reflux, nausea, bloating, discomfort in the chest and upper abdomen, and issues swallowing and expelling food from the throat and esophagus [10]. Obstructive symptoms such as vomiting, dysphagia, or pain or discomfort from the compression of nearby organs or tissues are more frequently linked to larger hiatal hernias [5], as in the case of our patient, in which vomiting and severe epigastric pain were his presenting symptoms. In extreme circumstances, ischemia in the herniated tissues and the requirement for immediate surgery may imprison massive hiatal hernias [5].

Challenges in diagnosis arise with epigastric pain, as a variety of cardiothoracic and intra-abdominal disorders could be responsible [11]. ECG, blood gases like lactate, and routine blood tests like cardiac enzymes and amylase can all aid in ruling out differential diagnosis [11], which were all insignificant in our patient. Imaging tests and clinical evaluation are usually used to diagnose a hiatal hernia [2]. Common methods for seeing the hernia, determining its size, and assessing related esophageal motility issues include upper gastrointestinal endoscopy, barium swallow radiography, and esophageal manometry [2]. Although upper endoscopy is usually advised, it frequently fails to show and appreciate the vast hiatal hernia, especially the stomach's organo-axial rotation [12]. In addition to the previously mentioned modalities, other radiological imaging for diagnosis includes chest radiography, which in our case demonstrated the presence of a hiatal hernia, and a CT scan of the abdomen, which was also performed on our patient and showed a large mixed type of hiatal hernia with the majority of the stomach sliding into the thoracic cavity.

Parker and Sabanathan reported the first case of sliding hiatal hernia complicated by incarceration, similar to our case, that had been diagnosed by CT scan and managed surgically through transhiatal esophagectomy performed by laparotomy [11]. The initial course of treatment for an esophageal hiatal hernia is typically conservative; however, in certain cases, surgery is strongly advised [13], and one of these situations that necessitates an urgent surgery is incarceration of the hernia, as in our case. A stomach perforation, severe bleeding, or gastrointestinal necrosis from strangulation could all be prevented by the surgical procedure [13].

Over the years, the fundamentals of giant paraesophageal hiatal hernia repair have not changed [14]. The main goals are reducing the hernia sac, performing extensive mediastinal dissection while maintaining the vagus nerves to guarantee sufficient intra-abdominal esophageal length, performing a gastroplasty only when required for extraesophageal length and following extensive mediastinal mobilization, repairing the crura while maintaining the crural peritoneal covering, and frequently performing a fundoplication [14].

## Conclusions

Strangulation of type III hiatal hernia, which includes both sliding and paraesophageal hernias, is a rare complication of this type and necessitates prompt surgical intervention to prevent the lethal outcomes if left untreated or if missed. Therefore, early diagnosis is essential to have better results and to improve the patient’s quality of life. Strangulation of a hiatal hernia must be kept in mind as a differential diagnosis for severe abdominal pain and other mentioned obstructive symptoms.
